# Supplementary material for: Patterns of Intron Gain and Loss in Fungi
Source: PLoS Biol. 2004 Nov 30;2(12):e422. doi: 10.1371/journal.pbio.0020422 (PMC532390; doi:10.1371/journal.pbio.0020422)
Supplement: Table S1 — Also available at http://genes.mit.edu/NielsenEtAl/. (4.3 MB ZIP). [file pbio.0020422.st001.zip › NielsenEtAl/html/129.html]

AN0228.1.NCU05194.1.MG04129.1.FG02470.1


```
 CLUSTAL W (1.82) Multiple Sequence Alignments - Introns Inserted


Sequence 1: MG04129.1	790 aa
Sequence 2: FG02470.1	1032 aa
Sequence 3: NCU05194.1	972 aa
Sequence 4: AN0228.1	915 aa
Alignment Length: 1067 aa
Number Identitical Residues: 418 aa
Alignment Score (without introns) 20684


MG04129.1 	------~---------------------------------------------------~-
NCU05194.1	------~---------------------------------------------------~-
FG02470.1 	MVTCDL0FARRSTNAMDNKLHRQALEYRAATFEVIEELEWFQLNSPSTRQGASPVLSE1K
AN0228.1  	------~---------------------------------------------------~-
          	                                                            

MG04129.1 	------------------------------------------------------------
NCU05194.1	-----------------MATPMAHSSDAGFMMSDGIASRGGATPRARNGHPSSSSARPRG
FG02470.1 	ASPTLLSIPYKDDRYYTTAAMATPSSDAGFMMSDGPS-RAAATPRRNYGVPSSSASRPRG
AN0228.1  	---------------------MSSLFDAVLQSELGSTAGSRDDRGLHSDALPSSRPQPMS
          	                      :   .:    . . :: .        .  .:: . . .

MG04129.1 	---------------------------------------------------------~--
NCU05194.1	PPSESLGAASEDEGEEGFADDQVP-VRSRPRNIADIPRVQDRIGVMVQRAFEAFIDG2YV
FG02470.1 	PPSENMGGAPSDEEGDGFADDQVP-RGLRINNTAEISRVEDRIGLLVQEHFESFIEN2FQ
AN0228.1  	ESNAPMSDANAFPDDQVPETSRSTSTRLRNPYVAGAPPVVDLAGEKVQQAFEELLEN~YQ
          	 ..   . :      .    .  .:        :  .   .  .   .   .   ..   

MG04129.1 	------------------------------------------------------------
NCU05194.1	ETPTSS---GQPTSSAVTTDKYYVAQIHGMRTYSLSTFYVDYKHVASWQDGTLAEAITNS
FG02470.1 	EDPISAPTPSAPTPSAVTTDKYYVAQIAGMRTYSLSTFYVDYRHLAAYENGSLADGVMRQ
AN0228.1  	EEAPPKSS-HTPQSSAPMTNKYYIAQIHGMAKWELSTLYVDFTHLTSLDNPILADAIANQ
          	. . . .:   . .::  :..   :.  .  . . ::   .    :: ..   :..   .

MG04129.1 	--------------MIAKYEPQYFREHRQPTTASSNMTTSGASNVGSASQSD------LA
NCU05194.1	YYRFLPFLTAALHSRIAAHEPQYFNEHRQPTASSAHSTTAASTRGANASQSD------FG
FG02470.1 	YYRFLPFLTAALHNMIAKYQPEYFREHRQPTTSS-NITTSAASHFGSSGQTES-----TH
AN0228.1  	YYRFQPFLVKALHNLIAKYEPEYFVSHRQATSSVSSQAGTSLMAGNSSVADDPKLDRTIR
          	     .    :  . ** ::*:** .***.*:: :  : :.     .:   :.. . :  

MG04129.1 	EKTTNQQTDKLFSIAFYNLPLVSRVRALRAKNIGQLLSISGTVTRTSEVRPELSLATFMC
NCU05194.1	IKNPNQQTDKLFAIAFYNLPLVSRVRSMRARNVGQLLAISGTVTRTSEVRPELALATFVC
FG02470.1 	RKNEHQQTDKLFSIAFYNLPLVSRVRALRATNIGQLLSISGTVTRTSEVRPELSMATFNC
AN0228.1  	EKTRHQQTDKLFALAFYNLPLVSRLRQLRTSQIGKLLSVSGTVTRTSEIRPELSLG----
          	 *. :*******::**********:* :*: ::*:**::*********:****::.    

MG04129.1 	QACKAIVPNVEQTFRYTEPTQCPNDNCQNRLAWQLDIRQSTFVD~WQKVRIQENSSEIPT
NCU05194.1	QACYAVVPNVEQTFRYTEPTQCPNLTCQNRTSWQLDIRQSTFVD~WQKVRVQENSSEIPT
FG02470.1 	EACRTVVPNVEQTFRYTEPTQCPNSTCQNRVAWQLDIRRSTFVD~WQKVRIQENSSEIPT
AN0228.1  	----------------------------------------TFIY1WQKVKLQESSHEIPT
          	                                        **:  ****::**.* ****

MG04129.1 	GSMPRTMDVIMRGEIVDRAKAGEKCVFTGALIVVPDVSQMGLPGLRATAVRDDK-APRPA
NCU05194.1	GSMPRTMDVILRGELVDRAKAGEKCIFTGALIVVPDVSQLGLPGVRNVSVRDDR----GA
FG02470.1 	GSMPRTMDVILRGEIVDRAKAGEKCIFTGALIVVPDVSQLGLPGLRPTAIRDDRNAPRGG
AN0228.1  	GSMPRTMDVILRGEMVDRVKAGERCIFTGTLIVIPDVSQLGLPGVRPEAVRDNS-GFRSN
          	**********:***:***.****:*:***:***:*****:****:*  ::**:  .    

MG04129.1 	EAGGSGVTGLKSLGVRDLTYRLAFLACMVIPHSDGLGGSSK----GGAAEIAAALAHAAS
NCU05194.1	DAGGSGVSGLKALGVRDLTYRLAFLACMVTPDVSAIGASGD----ALLMDIVGTLNSSAA
FG02470.1 	DAGGTGISGLKALGVRDLTYRLAFLACMVNPDTAASGQSAA----SSAADVVNALTTNTA
AN0228.1  	DVGGGGVSGLKALGVRDLTYRLAFLTCMVTPDTTTPGQQSNQQLSGQSNRILGSLNQNPD
          	:.** *::***:*************:*** *.    * .. .. :.    :  :*   . 

MG04129.1 	SDVTETQEEAQAAVLASYSTSEIDDLRSMVHSDKIYDRLVQSIAPTVYGHNVVKKGLLLQ
NCU05194.1	AETAETIKEAQEALLSSYTSAEMEDLRAMVHSDHIYARLVQSLAPTVYGHEVVKKGILLQ
FG02470.1 	NDIDQSVEDAQAAVLASMNPSEIDDLRAMVHGDHIYSRLVQSIAPMVYGHEVVKKGLLLQ
AN0228.1  	PEPDD--DKAQEAFLQSLSPAEVEDLKTMVHSEYIYSRLVDSMAPMIYGHRQIKKGLLLQ
          	 :  :  ..** *.* * ..:*::**::***.: ** ***:*:** :***. :***:***

MG04129.1 	LMSGVHKTT-AEGMQLRGDINICIVGDPSTSKSQFLK~YICSFAPRAVYTSGKASSAAGL
NCU05194.1	LLSGVSKTT-AEGMALRGDINICIVGDPSTSKSQFLK~YVVNFAPRAVYTSGKASSAAGL
FG02470.1 	LMSGVHKST-AEGMQLRGDINICIVGDPSTSKSQFLK~YVCSFAPRAVYTSGKASSAAGL
AN0228.1  	LVGGVAKSTEQESLQLRGDINICIVGDPSTSKSQFLK2YICSLHPRAVYTSGKASSAAGL
          	*:.** *:*. *.: ********************** *: .: ****************

MG04129.1 	TAAVVKDEETGEFTIEAGALMLADNG-ICAIDEFDKMDIADQVAIHEAMEQQTISIAKAG
NCU05194.1	TAAVVKDEETGEFTIEAGALMLADNG-ICCIDEFDKMDIADQVAIHEAMEQQTISIAKAG
FG02470.1 	TAAVVKDEETGEFTIEAGALMLADNG-VCAIDEFDKMDIADQVAIHEAMEQQTISIAKAG
AN0228.1  	TAKVVKDAETGEFTIEAGALMLANGGGICAIDEFDKMDISDQVAIHEAMEQQTISIAKAG
          	** **** ***************:.*.:*.*********:********************

MG04129.1 	IQATLNARTSILAAANPVGGRYNRKTTLRANINMSAPIMSRFDLFFVVLDECNERTDRHL
NCU05194.1	IQATLNARTSILAAANPVGGRYNRKTTLRANINMSAPIMSRFDLFFVILDECNEQVDRHL
FG02470.1 	IQATLNARTSILAAANPVGGRYNRKTTLRANINMSAPIMSRFDLFFVVLDECNEQVDRHL
AN0228.1  	IHTTLNARASILAAANPIGGRYNPKTTLRGNLNFSAPIMSRFDLFFVIRDEPNEDVDRNL
          	*::*****:********:***** *****.*:*:*************: ** ** .**:*

MG04129.1 	AEHIVGIHQLRDEAIEPEFSTEQLQRYIRFARTFRPEFTEEAKQTLVKHYRELRADDAQG
NCU05194.1	AEHIVGLHQNRDQAIEPEFSTEQLQRYIRFARTFRPEFTDEAKEVLVQRYKDLRADDAQG
FG02470.1 	AEHIVGIHQLRDEAVEPEFSTEQLQRYIRFAKTFRPEFTDEAKEVLVEKYKELRADDAQG
AN0228.1  	ADHIVNVHMNRDAAVEPEFSTEQLQRYIRFARTFRPVFREEAKAVLVEKYKELRANDAQG
          	*:***.:*  ** *:****************:**** * :*** .**::*::***:****

MG04129.1 	GVGKNSYRITVRQLESMIRLSEAIAKVNCVEEIASHMVDEAYNLLRQSIISVEHDDVEVD
NCU05194.1	GIGKNSYRITVRQLESMIRLSEAIAKANCVEEITPDFVNEAFHLLRQSIISVEHDDVEVD
FG02470.1 	GVGKNSYRITVRQLESMIRLSEAIAKVNCVEEISIDMVVEAYNLLRQSIISVEHDDVEVY
AN0228.1  	GMGRSSYRITVRQLESLIRLSEAIAKVNCVEEIVPKFVREAYDLLRQSIVTVEKDDVEVE
          	*:*:.***********:*********.******  .:* **:.******::**:***** 

MG04129.1 	DEEDA---PEDDQETLRRAAQAGQAP-------DEDSPMGGEEAAQN-ASTTTPASQKVT
NCU05194.1	DEEDAPEPPADSPEALQAAAAAASRVPEADMDGDGDVDMGGEAARQPSAAPSQAHREKQT
FG02470.1 	DEE-----PIEDSETLLRAADAAHGR-------DQEGDAVMEEDEQPGRSSVAPERKKQT
AN0228.1  	DDEG----AANADEDMPDRDR------------DGDSPMREEPQSAAAAEPVEP-RAKTK
          	*:*.    . :  * :                 * :     *     .  .  .   * .

MG04129.1 	ITHEKWVAMLNMMVQRVAEDEVSGSGEGISGDDLVNWYLEQKEDELASEEDFNREKKLAT
NCU05194.1	ISYDKYISIVNLIVSKVAEDETQGSGEGIEGEQLVEWYLEQKEEEMATEEEYHEERRLVG
FG02470.1 	ITYDNYVKMVNMFVQRIYEGEAAN-AEGIAGEDLVNWYLEQKEDELEGEEDYHREKALAN
AN0228.1  	ITYDKYMKILNLVVRRIREDEAQA-GEGVEQEDLLVWYLEQIEAELNNEEDLQRERSLAV
          	*:::::: ::*:.* :: *.*.   .**:  ::*: ***** * *:  **: :.*: *. 

MG04129.1 	MVLKKMVK0EHILMAVEAQSEDGDAATVEG--SSARAVRVVYVLHPNCAVEEI
NCU05194.1	KVLKRMVK0DNILMAIRGQGIVGEEGTVGEGTSARAEGNIVYVLHPNCAVEEF
FG02470.1 	MVLKKMVK~DNYLMALRGEGLDGEASSAS--------EQIVYIVHPNCSVEEH
AN0228.1  	KVLKRMVK~DNILMPIRGEGLVDEASEVQT-------DRTIYVLHPNCAIDEE
          	 ***:*** :: **.:..:.  .: . .          . :*::****:::*
```
